# Onycholysis Induced by Mycophenolate Mofetil in a Patient with Sjögren’s Syndrome and Scleritis

**DOI:** 10.51894/001c.144609

**Published:** 2025-09-30

**Authors:** Osman Muslehuddin

**Affiliations:** 1 Department of Internal Medicine Detroit Medical Center

## 104

### INTRODUCTION

The separation of the nail plate and nail bed is known as onycholysis, which has been associated with the use of certain immunosuppressants. In this case report, we describe the details of a 59-year-old female with Sjögren’s syndrome (SS) who developed bilateral onycholysis in her upper extremities three weeks after initiation of mycophenolate mofetil.

### CASE PRESENTATION

The 59-year-old female patient originally was referred to the rheumatology clinic for scleritis and complained of dry eyes, dry mouth, and recurrent rashes. Although initial autoimmune workup was negative, a biopsy of her salivary gland confirmed Sjögren’s syndrome.

Hydroxychloroquine was contraindicated because the patient had a history of retinal detachments and repairs, so she was started on mycophenolate mofetil. Within three weeks of beginning therapy, the patient complained of burning pain in her fingertips and nail bed swelling. She reported whitening of the free edge of her nails and detachment of her nail plate. The patient continued taking mycophenolate mofetil until her follow up the following month and these nail changes were documented. She was advised to stop the mycophenolate mofetil and at follow up after two weeks, these nail changes had significantly improved. Her nail changes were suspected to be due to the mycophenolate mofetil given the timeline of her changes and improvement off therapy.

### DISCUSSION

This case highlights onycholysis due to mycophenolate mofetil therapy, an important but rare adverse dermatological effect. Common causes of onycholysis include trauma to the nail, fungal/bacterial/yeast infections, chemical allergic reactions, iron deficiency, overactive thyroid, or adverse effects of chemotherapy or photosensitive medications.

Rheumatologists, dermatologists, allergists and other clinicians should consider mycophenolate mofetil as a cause of onycholysis in patients on immunosuppressants with otherwise unexplained nail changes.

### CONCLUSIONS

Mycophenolate mofetil should be considered as a cause of onycholysis. It is prudent to recognize this condition early and recommend discontinuation to prevent further worsening of nail changes. More studies are essential to confirm this relationship and better define risk factors of this adverse effect.

### .

